# Production of Reverse Transcriptase and DNA Polymerase in Bacterial Expression Systems

**DOI:** 10.3390/bioengineering11070727

**Published:** 2024-07-18

**Authors:** Kristína Hriňová, Johana Dlapová, Bohuš Kubala, Ľubica Kormanová, Zdenko Levarski, Eva Struhárňanská, Ján Turňa, Stanislav Stuchlík

**Affiliations:** 1 Department of Molecular Biology, Faculty of Natural Sciences, Comenius University in Bratislava, 84215 Bratislava, Slovakia; hrinova10@uniba.sk (K.H.); dlapova2@uniba.sk (J.D.); lubica.kormanova@uniba.sk (Ľ.K.); struharnans1@uniba.sk (E.S.); jan.turna@uvp.uniba.sk (J.T.); stanislav.stuchlik@uniba.sk (S.S.); 2 Laboratory for Microbial Ecology, Institute of Molecular Biology, Slovak Academy of Sciences, Dúbravská Cesta 21, 84551 Bratislava, Slovakia; bohus.kubala@savba.sk; 3 Science Park, Comenius University in Bratislava, 84104 Bratislava, Slovakia; 4 ReKoMBe, s.r.o., 84102 Bratislava, Slovakia

**Keywords:** *Vibrio natriegens*, *Escherichia coli*, reverse transcriptase, DNA polymerase, protein production, extracellular production

## Abstract

DNA amplification and reverse transcription enzymes have proven to be invaluable in fast and reliable diagnostics and research applications because of their processivity, specificity, and robustness. Our study focused on the production of mutant Taq DNA polymerase and mutant M-MLV reverse transcriptase in the expression hosts *Vibrio natriegens* and *Escherichia coli* under various expression conditions. We also examined nonspecific extracellular production in *V. natriegens*. Intracellularly, M-MLV was produced in *V. natriegens* at the level of 11% of the total cell proteins (TCPs) compared with 16% of TCPs in *E. coli*. We obtained a soluble protein that accounted for 11% of the enzyme produced in *V. natriegens* and 22% of the enzyme produced in *E. coli*. Taq pol was produced intracellularly in *V. natriegens* at the level of 30% of TCPs compared with 26% of TCPs in *E. coli*. However, Taq pol was almost non-soluble in *E. coli*, whereas in *V. natriegens*, we obtained a soluble protein that accounted for 23% of the produced enzyme. We detected substantial extracellular production of Taq pol. Thus, *V. natriegens* is a suitable alternative host with the potential for production of recombinant proteins.

## 1. Introduction

The Gram-negative bacterium *Escherichia coli* remains one of the most widely used expression systems and continues to be the organism of choice in both laboratory experiments and early-stage commercial developments. Its longstanding dominance persists because of its versatility and reliability, making it the preferred platform for initial experimentation in comparison with other expression systems. The growth conditions for *E. coli* cultures are cost-effective, and its genetics are extensively understood, enabling the construction of various variants and strains with standard molecular techniques. However, some limitations are inherent to this system, including low expression levels, inclusion body formation, and protein inactivity. The doubling time of *E. coli* is approximately 20 minutes in rich medium, indicating room for improvement in its growth and the creation of new strains. However, this approach also has its limitations, and research is ongoing [1]. Nevertheless, the most effective solution might be the implementation of novel alternative hosts that offer unique features and additional advantages.

*Vibrio natriegens* is a Gram-negative marine bacterium that is considered to be the fastest-growing host organism to date, with a generation time of 7–10 min [2]. It tolerates a wide pH range, its pH optimum is 7.5, and its optimum temperature cultivation in BHI medium is 37 °C [3]. A significant milestone was reached in 2016, marked by the independent publication of two papers [4,5], which were well received by the biotechnology community and which created strong interest in this organism. Its genome consists of two chromosomes of 3,248,023 bp and 1,927,310 bp, respectively, that together encode 4578 open reading frames; hence, the total genome size is approximately 5.17 Mb, i.e., over 0.5 Mb larger than the *E. coli* genome [4]. *V. natriegens* can utilize a wide range of substrates and grow robustly on sucrose [5], a feedstock that is both economically and environmentally beneficial. This stands in contrast to the majority of existing industrial *E. coli* strains, which are unable to utilize sucrose [6]. Moreover, at 37 °C, the generation time of *V. natriegens* is 14.8 min in LB3, which is 2.1 times faster than that of *E. coli* in LB (31.3 min). These results confirm *V. natriegens* as the fastest known, free-living organism. With the development of various genetic tools, we can, therefore, now use this organism as an alternative host for protein production. Several different proteins have also been produced in this system, making it a suitable alternative to *E. coli* [7,8,9,10,11,12]. Rapid growth, low cost, and high production yield are important factors that make this host organism attractive for industrial production.

During the production of recombinant proteins, a soluble protein can be obtained in several ways, including the lowering of the cultivation temperature, a change in the host organism, or even extracellular production. Such production might be more advantageous than intracellular production because we can produce toxic proteins, proteins with preserved biological activity, and correctly folded proteins with this strategy, and such production simplifies downstream processes and overall costs. Penicillin-binding proteins (PBPs) are proteins that play an important role in cell wall peptidoglycan synthesis [13]. D-alanyl-D-alanine (D,D)-carboxypeptidases are low molecular weight PBPs that play a significant role in maintaining normal cell morphology [14]. Overexpression of D,D-carboxypeptidases might result in the disruption of the cell wall structure, resulting in the extracellular production of the target protein. Such production has been achieved not only in *E. coli* [15], but also in *V. natriegens* [16].

Taq DNA polymerase, isolated from *Thermus aquaticus*, is a widely utilized thermostable enzyme known for its thermostability and its role in DNA amplification techniques [17]. Originally isolated in 1976 from samples taken from Yellowstone National Park, USA, Taq DNA pol consists of 832 amino acids, with a molecular weight of approximately 94 kDa and exhibits a specific activity of approximately 292,000 U/mg [18]. It has three domains, with the N-terminal domain exhibiting exonuclease activity and the C-terminal domain being responsible for polymerase activity [19]. Taq DNA pol replicates a 1 kbp DNA strand in under 10 s at 72 °C [20] and has a high fidelity, with an error rate estimated to be between 3 × 10^−4^ and 3 × 10^−6^ errors per polymerized nucleotide [21]. This enzyme significantly improves yield, specificity, and automation in PCR applications [22]. Additionally, protein engineering techniques have been used to create mutant Taq DNA pol variants, such as those sensitive to cold or with enhanced functionality, offering specific solutions for various research needs [23,24,25,26].

The reverse transcriptases are a special group of DNA polymerases and have three enzymatic activities: (1) they employ RNA as a template for the synthesis of a complementary DNA strand, generating an RNA/DNA hybrid molecule (RNA-dependent DNA polymerase activity, RDDP); (2) they can use a DNA template to create a complementary DNA strand (DNA-dependent DNA polymerase activity, DDDP); (3) they degrade RNA strands annealed to DNA (RNase H activity). Such transcriptases are important enzymes in the fields of molecular biology, genetics, and medicine. Laboratory research is thus focused on the availability of efficient and functional reverse transcriptases with various mutations and improved properties and their subsequent production for various biotechnological applications, such as RT-PCR, qPCR, cDNA cloning, and RNA sequencing. Reverse transcriptase M-MLV was discovered in 1970 by Edward Scolnick [27] at the beginning of the boom in reverse transcriptase studies. M-MLV reverse transcriptase, unlike most reverse transcriptases, is an enzyme with only one subunit. Its molecular mass is approximately 75 kDa, and its length is 671 amino acids. It shares a similar structure with other DNA polymerases in this family, as it consists of three separate domains: palm, thumb, and fingers. In addition, as a reverse transcriptase with RNase H activity, it contains an RNase H domain with its own active site and a terminal domain. The crystal structure of the N-terminal fragment was solved by Georgians et al. in 1995 [28], and the structure of the whole enzyme was clarified in 2004 by Das and Georgiadis [29].

In this study, we focus on *V. natriegens* strains under various growth conditions in comparison with *E. coli*. We introduce *V. natriegens* as an attractive expression host for the production of industrially relevant proteins, namely Taq DNA polymerase and M-MLV reverse transcriptase. Advanced and innovative expression systems have the potential to drive down further the production costs of enzymes.

## 2. Materials and Methods

### 2.1. Bacterial Strains and Plasmids

Table 1 provides a comprehensive list of all the strains used here. We utilized two strains of *V. natriegens*. The first was the genetically engineered Vmax™ Express strain of *V. natriegens*, featuring an IPTG-inducible T7 RNA polymerase cassette and a knockout of extracellular nucleases for enhanced gene expression. For comparisons, we employed a strain of *E. coli* BL21(DE3) containing the same cassette. The second was the strain of *V. natriegens* (*V. natriegens* PF) engineered by Pfeifer et al. [30] to contain deletions in predicted prophage loci, impacting its robustness and production stability. *V. natriegens* PF lacks a T7 polymerase cassette, and so for comparison with *E. coli*, we used the BL21 strain, which is a widely used non-T7 expression *E. coli* strain.

### 2.2. Construction of Recombinant Plasmids

Mutant M-MLV reverse transcriptase was amplified by PCR from the pUC57 plasmid with a commercially synthesized gene for mutant M-MLV (GenScript, Piscataway, NJ, USA). The point mutations were designed according to Baranauskas et al. [31] and provide higher processivity and thermostability of this enzyme. We inserted sequences for restriction sites of chosen restrictases by specific primers on both sides of the gene coding for M-MLV to be able to insert it in the right orientation into the pJexpress404 vector (Fwd_M-MLV: AAAACATATGATGGCGGGTACGTTGAACATCGATGAACATCGCC, Rev_M-MLV: AAAACTCGAGCAGCAGCGTGCTTGTATCCGGTGTCTCATAATCGC). After PCR amplification of M-MLV, the PCR product and pJexpress404 were digested with NdeI and XhoI (New England Biolabs^®^) and ligated by T4 DNA Ligase (New England Biolabs^®^). Mutant Taq DNA polymerase was cloned from the pUC57 plasmid with a commercially synthesized gene for mutant Taq pol (GenScript). The point mutations were designed as given in the literature [24,25] in order to provide a cold-sensitive mutant with tolerance to various PCR inhibitors. Recombinant expression plasmid was prepared by digestion of pUC57-mutTaqDNApol and pET28a with the restriction endonuclease enzymes BamHI and Hind III (New England Biolabs^®^), with subsequent ligation by T4 DNA Ligase (New England Biolabs^®^). Both recombinant plasmids were verified by sequencing, and we transformed both plasmids into *E. coli* and *V. natriegens* strains.

### 2.3. Transformation

Strains Vmax™ Express and BL21(DE3) were transformed with the designed plasmid pET28a—mutTaqDNApol (Table 2). BL21 and PF were transformed with the designed plasmid pJexpress404-mutM-MLV reverse transcriptase (Table 2). Chemically competent cells BL21(DE3) and BL21 were prepared in advance by using CaCl_2_ and MgCl_2_ treatment. Cells were cultured to OD600 = 0.4–0.5. After this optical density (OD) was reached, cells were chilled on ice for 10 min. The culture was centrifuged at 2000× *g*, 4 °C, for 10 min, and the sediment was gently suspended in 5 mL of 100 mM iced MgCl_2_. The suspension was centrifuged again under the same conditions, and the obtained sediment was resuspended in 1.5 mL of 100 mM iced CaCl_2_ and incubated on ice for 1.5 h. After incubation, 500 µL of 50% glycerol (*v*/*v*) was added, and the suspension was aliquoted. Plasmid DNA (100–150 ng) was added to a 50 µL aliquot of cells, after which the mixture was subjected to a heat shock at 42 °C for 45 s, followed by a chilling step on ice. After the addition of LB growth medium, the mixture was incubated at 37 °C for 90 min. Transformed cells were plated on LB agar plates with glucose and the respective antibiotic (100 µg/mL ampicillin, 50 µg/mL kanamycin) and incubated overnight. For the transformation of *V. natriegens* strains, we opted for a modified electroporation protocol as described by Weinstock et al. [5]. *V. natriegens* cells were grown in BHIv2 medium to OD600 = 0.5. The bacterial culture was incubated on ice for 15 min. Cells were harvested at 4000× *g* at 4 °C for 10 min. The bacterial pellet was washed with electroporation buffer (680 mM sucrose, 7 mM K_2_HPO_4_, pH 7.2) and centrifuged at 4000× *g* at 4 °C for 10 min, for a total of three times. The pellet was resuspended in residual electroporation buffer, and the volume was adjusted to OD600 = 19. The mixture was aliquoted and stored at −80 °C. After the addition of 50–200 ng plasmid DNA to the cells, the mixture was transferred to an electroporation cuvette with a gap width of 0.1 cm (Bio-Rad). For the transformation, we used the following parameters: 800 V, 25 µF, 200 Ω (Bio-Rad). Following transformation, cells were recovered in LB3 medium supplemented with 680 mM sucrose and shaken at 28 °C for at least 2 h. The mixture was then spread on the LB3 agar plates enriched with 100 μg/mL ampicillin and 1% sucrose. The plates were cultivated at 37 °C overnight.

For the two-plasmid system, we first transformed the cells with an expression plasmid and then prepared competent cells from the recombinants, which were subsequently transformed with a second plasmid (pRSFDuet-T5/T7-PBP5/6).

### 2.4. Protein Production in Erlenmeyer Flasks

An overnight culture of *E. coli* BL21 (DE3)/BL21 and two strains of *V. natriegens* (Vmax™ Express/PF) cells carrying a plasmid with mutant Taq DNA polymerase/M-MLV reverse transcriptase was inoculated at a ratio of 1:100 into 50 mL of LB medium with the addition of 0.1% glucose (*v*/*v*) or 1% glycerol (*v*/*v*). The cell culture was allowed to grow to give OD600 = 0.5–1 when using LB/LB3 medium and OD600 = 2 when using the richer TB medium (with the addition of 1.5% NaCl (*w*/*v*)). OD was measured using a SmartSpec^TM^ Plus instrument (Bio-Rad, Hercules, CA, USA). We induced expression by adding the inducer IPTG at a final concentration of 1 mM. Expression was carried out for 4 h with sampling every hour, with all samples being diluted to equal OD600 values. We centrifuged the samples for 3 min at 12,000× *g* on a MiniSpin centrifuge (Eppendorf, Hamburg, Germany), and the cell cultures obtained after 4 h from the start of expression induction were centrifuged for 10 min, 4 °C at 7690× *g* (UNIVERSAL 320R, Hettich, Tuttlingen, Germany).

### 2.5. Cell Disruption

We resuspended the pellet obtained after the shaking flask expression in a 15 mL homogenizer buffer solution. We used three different solutions, the compositions of which are listed in Table 3. Subsequently, we sonicated the cells by using the Sono puls device (Bandelin) for 8 min and 15 s, with pulses of 15 s and cooling intervals of 45 s at an amplitude of 30%. The cell suspension was then centrifuged at 7690× *g* for 15 min at 4 °C. We analyzed the pellet and supernatant using SDS-PAGE and Western blot.

### 2.6. SDS-PAGE Electrophoresis

For the electrophoretic separation of proteins under denaturing conditions in polyacrylamide gel, we carried out SDS-PAGE according to Laemmli et al. [32] within the apparatus from Hoefer™ (Mighty Small™ II Mini Vertical Electrophoresis Systems, MA, USA). We prepared a 12% separation gel and a 4% application gel. After assembling the apparatus, we applied an 8 μL sample and 3.5 μL size standard PageRuler^TM^ Prestained Protein Ladder 26616 (Thermofisher, Waltham, MA, USA) or Novex™ Sharp Pre-stained Protein Standard (Invitrogen, Waltham, MA, USA). Electrophoretic separation was run in 1× concentrated electrode buffer solution. We set the voltage to the maximum, with the current set to 20 mA per gel for 90 min. After electrophoretic separation, we stained the gel with a staining solution (Coomassie brilliant blue G-250 0.1%, ammonium sulfate 10%, phosphoric acid 2%).

### 2.7. Western Blot

Western blot was used for the specific detection of mutant Taq DNA polymerase and mutant M-MLV reverse transcriptase. Protein samples separated on SDS-PAGE were transferred to a PVDF membrane (Bio-Rad). The semi-dry transfer was performed using a Biometra blotter. The parameters of the analysis were set to 10 V, 220 mA, 45 min. The PVDF membrane was incubated in a specific antibody solution (mouse anti-His) (Invitrogen) at a dilution of 1:1000 in a 1% skimmed milk solution at 4 °C overnight. The next day, the membrane was washed with TBST solution and incubated with anti-mouse secondary antibody solution (1:10,000) in a 1% skimmed milk solution (Goat Anti-Mouse IgG, Sigma Aldrich, Saint-Louis, MO, USA) for 1 h at room temperature. Proteins on the membrane were visualized by using a chemiluminescence solution, namely Clarity Max Western ECL Substrate kit (Bio-Rad), according to the manufacturer’s protocol. Subsequently, the signal was detected by using an ImageQuant LAS 500 chemiluminescence CCD camera (GE Healthcare, Chicago, IL, USA).

### 2.8. Protein Precipitation

The sample containing mutant Taq DNA polymerase was initially heated at 70 °C for 20 min to remove thermolabile proteins. After this heating step, we centrifuged the suspension at 12,000× *g* at 4 °C (UNIVERSAL 320R, Hettich) and then precipitated the protein by using a modified protocol according to Pluthero [33] with ammonium sulfate. We added (NH_4_)_2_SO_4_ to the sample at a concentration of 176 g/L and mixed it on ice for 1 h. After centrifuging the suspension, we resuspended the sediment in a sonication buffer A and dialyzed it.

### 2.9. Dialysis

To remove low molecular weight substances from the sample after protein precipitation and affinity chromatography, we chose dialysis. The sample was transferred into a SnakeSkin Dialysis Tubing bag, 10K MWCO, 16 mm (Thermo Fisher Scientific, Waltham, MA, USA), and dialyzed against sonication buffer A. The dialysis was performed at 4 °C with constant stirring.

### 2.10. Affinity Chromatography

Purification of the samples was carried out by high-pressure affinity chromatography on a ÄKTA Avant 25 (GE Healthcare) with 1 mL HisTrap^TM^ HP (GE Healthcare), which contained Ni Sepharose^TM^ affinity medium for the capture and purification of affinity-tagged histidine proteins (6× His-tag). The column was washed with water and equilibration buffer (50 mM Tris HCl, pH 8.0, 0.5 M NaCl). The sample was applied to the column via a pump, and the uncaptured fraction was collected by an automated fraction collector. The bound proteins on the column were eluted using an elution solution (50 mM Tris HCl pH 8.0, 0.5 M NaCl, 0.5 M imidazole). The fraction containing the elution was dialyzed against sonication buffer A.

### 2.11. Reverse Transcription

RNA encoding green fluorescent protein (GFP) at a concentration of 50 mg/L was used as the template. The positive control was a sample containing commercial reverse transcriptase from the QuantiFast SYBR Green RT-PCR Kit. Reverse transcription was carried out in a LabCycler BASIC, SensoQuest at 42 °C for 60 min.

### 2.12. PCR

The RNA transcribed into cDNA was amplified by PCR, which was performed in a Labcycler Gradient Thermocycler (SensoQuest, Göttingen, Germany). The protocol specified by the manufacturer of the Taq DNA Polymerase (New England Biolabs^®^) (denaturation at 95 °C for 20 s, annealing at 55 °C for 30 s, and polymerization at 68 °C for 60 s, in 30 cycles) was used for amplification.

## 3. Results

### 3.1. Protein Production

**Figure 1:**
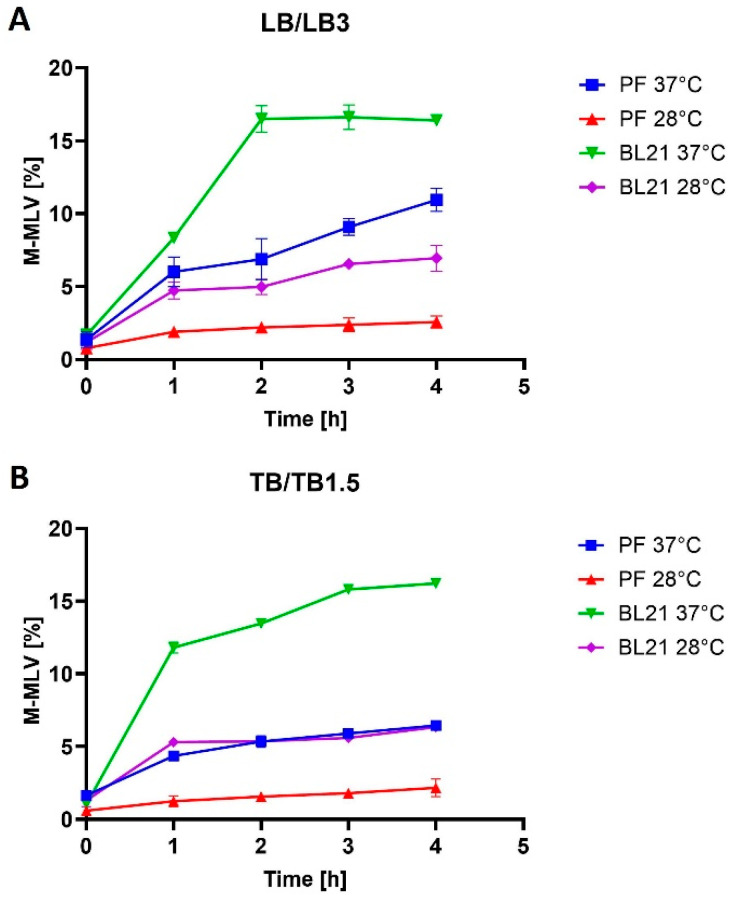
M-MLV production comparison. (**A**) illustrates the amount of mutant M-MLV reverse transcriptase produced from TCPs (in %) in LB/LB3 media at various temperatures during production in *E. coli* BL21 and *V. natriegens* PF. (**B**) illustrates the amount of mutant M-MLV reverse transcriptase produced from TCPs (in %) in TB/LB1.5 media at various temperatures during production in *E. coli* BL21 and *V. natriegens* PF.

We conducted a comparison of protein production of mutant Taq DNA polymerase and mutant M-MLV reverse transcriptase in *E. coli* and *V. natriegens* under identical conditions (Figure 1A,B). This experiment involved using the same media (LB/LB3, TB/TB1.5), with the addition of 3% NaCl in the LB medium and 1.5% NaCl in the TB medium for *V. natriegens*, as NaCl is essential for its optimal growth. We also tested protein production under two different temperatures: 37 °C and 28 °C. Expression in 50 mL Erlenmeyer flasks showed that the expression rate in all cases was influenced by lowering the temperature and by using the different media.

**Figure 2:**
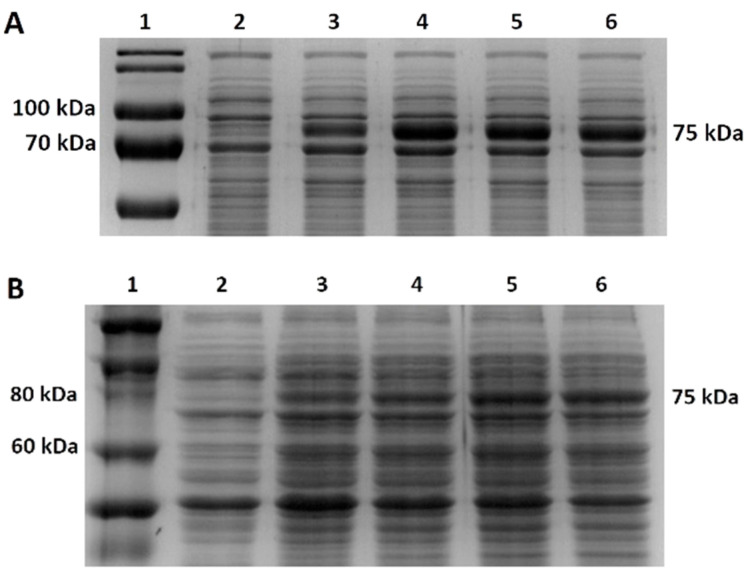
M-MLV protein production in Erlenmeyer flasks. (**A**) SDS-PAGE analysis of protein production of mutant M-MLV reverse transcriptase in LB medium at 37 °C in *E. coli* BL21 cells. 1—molecular standard; 2—0 h/before induction; 3—1 h after induction with 1 mM IPTG; 4—2 h after induction; 5—3 h after induction; 6—4 h after induction. (**B**) SDS-PAGE analysis of protein production of mutant M-MLV reverse transcriptase in LB3 medium at 37 °C in *V. natriegens* PF cells. 1—molecular standard; 2—0 h/before induction; 3—1 h after induction with 1 mM IPTG; 4—2 h after induction; 5—3 h after induction; 6—4 h after induction.

*V. natriegens* PF cells and *E. coli* BL21 cells transformed with pJexpress404M-MLV were induced with 1 mM IPTG to produce a mutant M-MLV reverse transcriptase. Depending on the densitometric analysis of the SDS-PAGE gels (Figure 2A,B shows the production of M-MLV in PF and BL21 under optimal conditions), we calculated the relative rate of M-MLV of the total cell proteins (TCPs). The target protein accounted for approximately 16% of TCPs by cultivation of BL21 cells in LB medium at 37 °C after 4 h. For expression at 28 °C, the target protein accounted for nearly 7% of TCPs. During the cultivation of PF cells in LB3 medium at 37 °C, the target protein reached 16% of TCPs after 4 h. For expression at 28 °C, the target protein reached only 2.5% of TCPs after 4 h. Cultivation in TB/TB1.5 medium showed a similar result. M-MLV production in BL21 cells was 2.5 times higher than in PF cells at 37 °C and 2.9 times higher at 28 °C.

**Figure 3:**
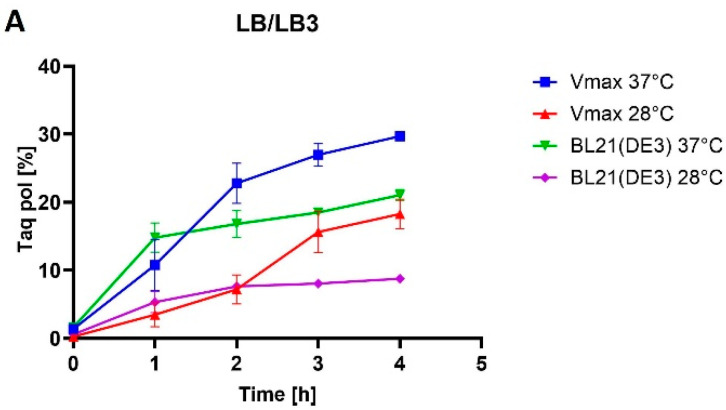

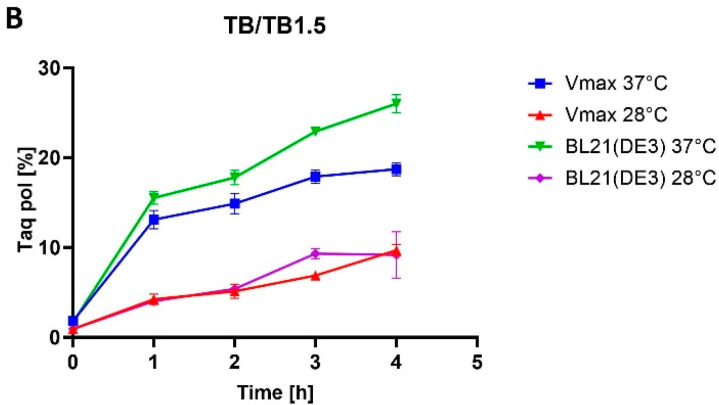
Taq pol production comparison. (**A**) illustrates the amount of mutant Taq DNA polymerase produced from TCPs (in %) in LB/LB3 media at various temperatures during production in *E. coli* BL21(DE3) and *V. natriegens* Vmax™ Express. (**B**) illustrates the amount of mutant Taq DNA polymerase produced from TCPs (in %) in TB/TB1.5 media at various temperatures during production in *E. coli* BL21(DE3) and *V. natriegens* Vmax™ Express.

**Figure 4:**
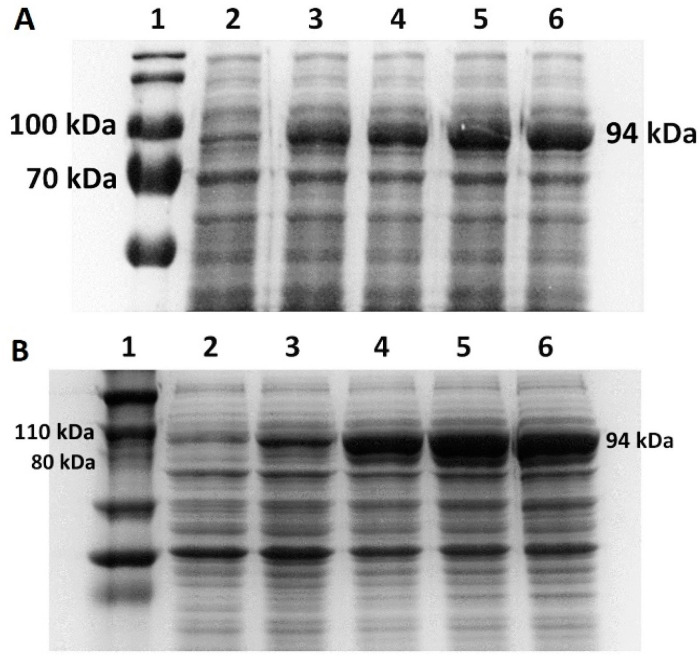
Taq pol protein production in Erlenmeyer flasks. (**A**) SDS-PAGE analysis of protein production of mutant Taq DNA polymerase in LB medium at 37 °C in *E. coli* BL21(DE3) cells. 1—molecular standard; 2—0 h/before induction; 3—1 h after induction with 1 mM IPTG; 4—2 h after induction; 5—3 h after induction; 6—4 h after induction. (**B**) SDS-PAGE analysis of protein production of mutant Taq DNA polymerase in LB3 medium at 37 °C in *V. natriegens* Vmax™ Express. 1—molecular standard; 2—0 h/before induction; 3—1 h after induction with 1 mM IPTG; 4—2 h after induction; 5—3 h after induction; 6—4 h after induction.

*V. natriegens* Vmax™ Express cells and *E. coli* BL21(DE3) cells transformed with pET28a-mutTaqDNApol were induced with 1 mM IPTG to produce a mutant Taq DNA polymerase (Figure 3A,B). Our experiments revealed that, when using LB/LB3 media, the production of Taq DNA polymerase in BL21(DE3) (Figure 4A) and Vmax™ Express (Figure 4B) was more effective in the case of Vmax™ Express (Figure 3A). After 4 h of expression, the target protein accounted for approximately 30% of TCPs at 37 °C, whereas at 28 °C, it constituted nearly 20% of TCPs. Therefore, the production of the target protein was 1.5 times higher in the *V. natriegens* strain at 37 °C in LB medium and 2 times higher at 28 °C compared with *E. coli*. As shown in Figure 3B, the use of TB media led to a higher production of the target protein in BL21(DE3), reaching approximately 26% of TCPs after 4 h, compared with approximately 19% of TCPs in Vmax™ Express. However, production at lower temperatures in TB media led to approximately the same production of the target protein in both strains, resulting in approximately 10% of the target protein of TCPs.

### 3.2. Solubility

**Figure 5:**
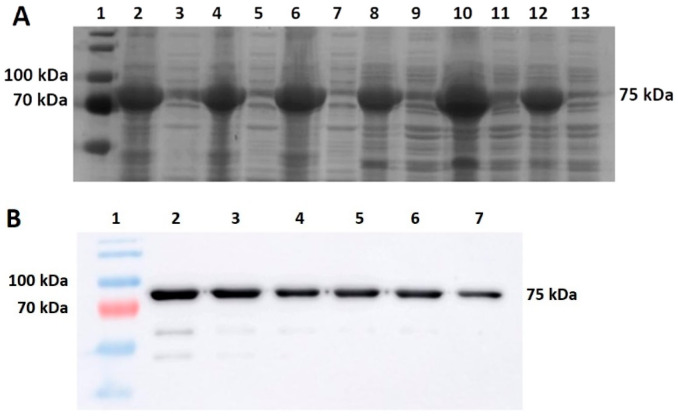
M-MLV solubility analysis. (**A**) SDS-PAGE analysis of insoluble and soluble fractions of M-MLV in *E. coli* BL21 (lanes 2–7) and *V. natriegens* PF (8–13) in various sonication buffers (same order as shown in Figure 6). (**B**) Western blot analysis of soluble fractions of M-MLV in *E. coli* BL21 (lanes 2–4) and *V. natriegens* PF (5–7) in various sonication buffers (same order as shown in Figure 6).

**Figure 6:**
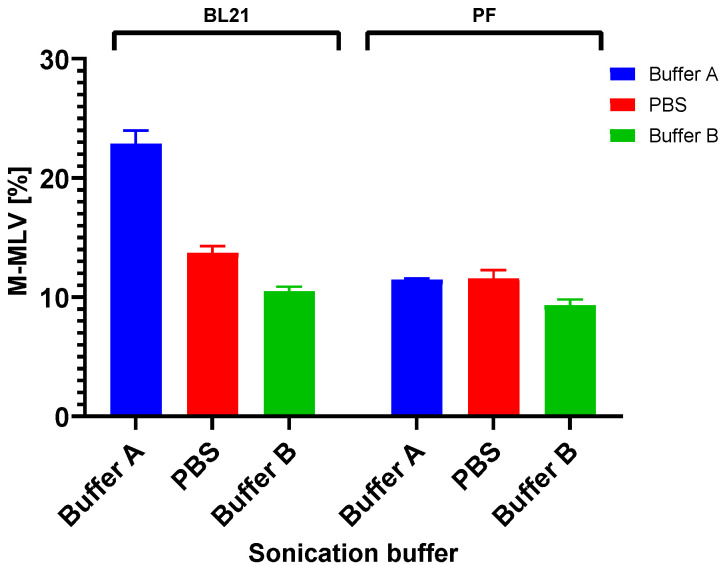
M-MLV solubility comparison. Soluble fractions of M-MLV in *E. coli* BL21 and *V. natriegens* PF in various sonication buffers (buffer compositions in Table 2).

Another parameter of interest to us was the solubility of the target protein in various sonication buffers. To assess solubility in the different buffers, we harvested cells after 4 h of expression in LB/LB3 medium with both strains of *E. coli* and *V. natriegens*. We were able to compare the influence of the production organism on protein solubility and the effects of the buffers on protein solubility. A lowering of the temperature did not increase the solubility of the protein. One approach to increasing solubility is the use of a range of sonication buffers. After M-MLV production in PF/BL21 cells in LB3/LB medium at 37 °C, the cells were homogenized in various sonication buffers (Figure 5A). Buffers A and B and PBS were used in both PF and BL21 cells for comparison. The highest amount of the M-MLV soluble fraction was found in BL21 cells in Buffer A (Figure 6) when M-MLV reached almost 23% of TCPs, which is almost two times higher than in PF cells. In buffer PBS, this value was approximately 14% (BL21) compared with PF, for which the soluble fraction of M-MLV reached approximately 12% of TCPs. A similar result was obtained in Buffer B: nearly 11% compared to 9% of TCPs. For confirmation of the SDS-PAGE analysis, we also performed Western blot analysis (Figure 5B).

**Figure 7:**
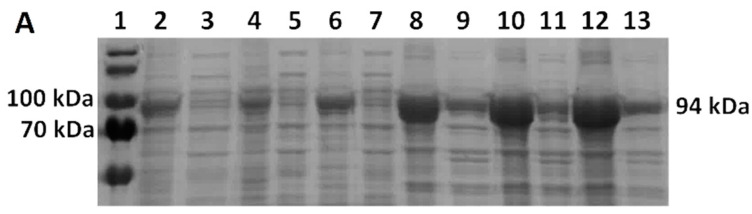

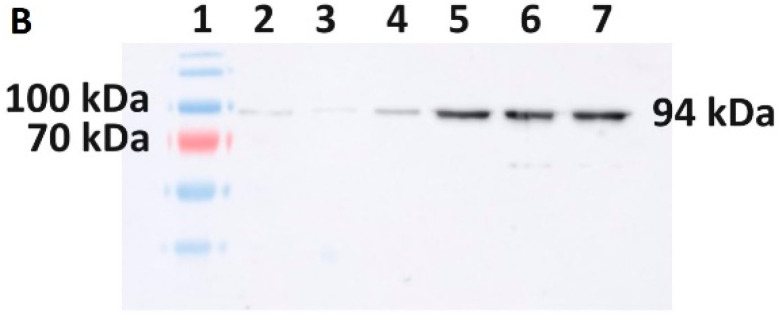
Taq pol solubility analysis. (**A**) SDS-PAGE analysis of insoluble and soluble fractions of Taq DNA polymerase in *E. coli* BL21(DE3) (lanes 2–7) and *V. natriegens* Vmax™ Express (8–13) in various sonication buffers (same order as shown in Figure 8). (**B**) Western blot analysis of soluble fractions of Taq DNA polymerase in *E. coli* BL21(DE3) (lanes 2–4) and *V. natriegens* Vmax™ Express (5–7) in various sonication buffers (same order as shown in Figure 8).

**Figure 8:**
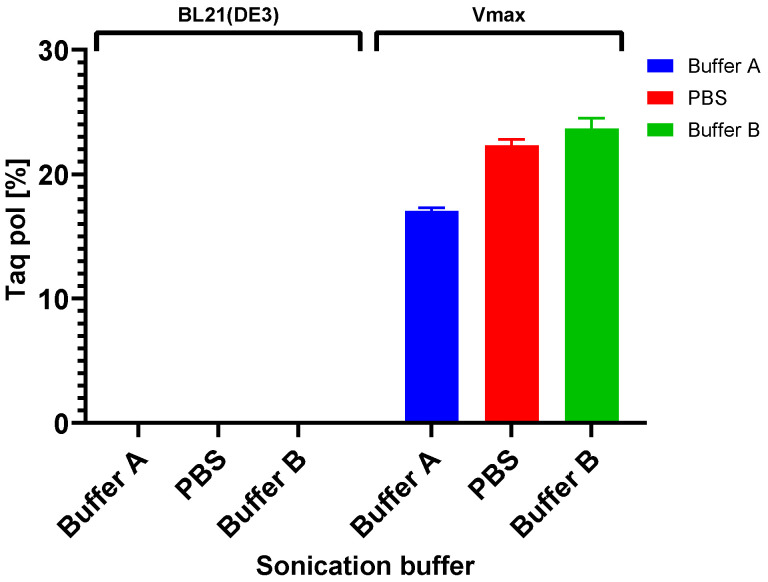
Taq pol solubility comparison. Soluble fractions of Taq pol in *E. coli* BL21(DE3) and *V. natriegens* Vmax™ Express in various sonication buffers (buffer compositions in Table 2).

We analyzed the solubility of Taq DNA polymerase by SDS-PAGE (Figure 7A) and evaluated it with GelAnalyzer^TM^. Partial solubility was achieved when using all mentioned buffers during production in Vmax™ Express, ranging from 17% in Buffer A to 23% in Buffer B (Figure 8). However, as seen in Figure 7A, a comparison of soluble and insoluble fractions of BL21(DE3) and Vmax™ Express shows no apparent soluble protein fraction during production in *E. coli*. However, Western blot analysis (Figure 7B) reveals that small amounts of soluble proteins are present and that the use of Buffer B leads to the highest solubilization despite only an insignificant amount of protein being soluble.

### 3.3. Extracellular Production in V. natriegens

**Figure 9:**
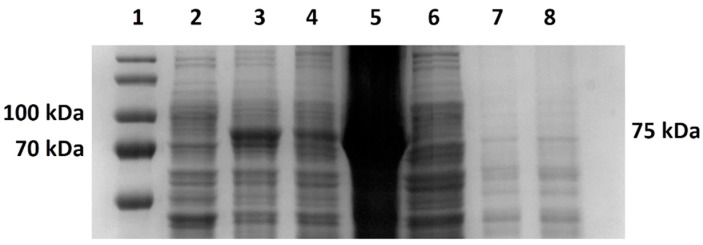
M-MLV extracellular production in *V. natriegens*. SDS-PAGE analysis of extracellular production of mutant M-MLV reverse transcriptase in LB3 medium at 37 °C in PF cells. Lanes 2–4 represent intracellular production, lanes 7, 8 represent extracellular production. 1—molecular standard; 2—0 h before induction; 3—4 h after induction with 1 mM IPTG; 4—24 h after induction; 5—insoluble fraction after 24 h; 6—soluble faction after 24 h; 7—extracellular production without co-expression after 24 h; 8—extracellular production with co-expression after 24 h.

**Figure 10:**
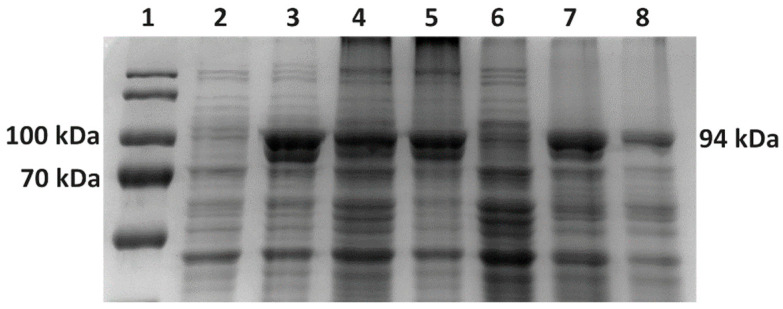
Taq pol extracellular production in *V. natriegens*. SDS-PAGE analysis of extracellular production of mutant Taq DNA polymerase in LB3 medium at 37 °C in Vmax™ Express cells. Lanes 2–4 represent intracellular production, lanes 7, 8 represent extracellular production. 1—molecular standard; 2—0 h before induction; 3—4 h after induction with 1 mM IPTG; 4—24 h after induction; 5—insoluble fraction after 24 h; 6—soluble faction after 24 h; 7—extracellular production without co-expression after 24 h; 8—extracellular production with co-expression after 24 h.

Another way of obtaining a soluble protein is by producing protein in the growth medium. Overproduction of selected low-molecular-weight D,D-carboxypeptidases affects cell permeability and membrane integrity and increases extracellular protein leakage into the growth medium in *V. natriegens* without the use of secretion signals, etc. [16]. Extracellular production of M-MLV was tested in LB3 medium at 37 °C for 24 h. However, no extracellular production of this protein was observed by SDS-PAGE analysis (Figure 9). For M-MLV reverse transcriptase, intracellular production appeared to be more effective. Extracellular production of Taq DNA polymerase (Figure 10) was tested in LB3 medium, at 37 °C, for 24 h to achieve the highest possible amount of extracellularly produced protein. However, as seen in lane 4 in Figure 10, partial degradation of the target protein occurs after 24 h following induction compared with lane 3 (4 h after induction) when the protein was produced intracellularly. Interestingly, higher extracellular production was observed after 24 h without co-expression (lane 7 in Figure 10), with 25% leaked TCPs compared with 22% leaked TCPs with co-expression (lane 8 in Figure 10).

### 3.4. Verification of Activity

**Figure 11:**
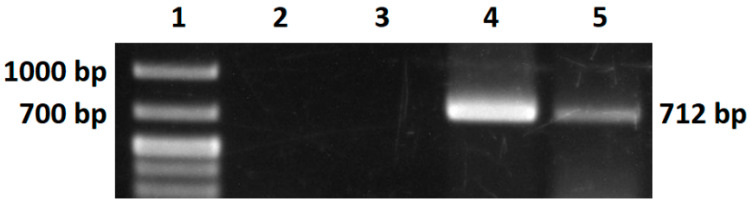
Activity verification. Agarose gel electrophoresis of PCR. 1—molecular standard, 2—negative control from PCR, 3—negative control from reverse transcription, 4—positive control, 5—tested sample with M-MLV.

The soluble fraction of the protein from intracellular production was purified by affinity chromatography by using a 1 mL Ni^2+^ column. Verification of activity in M-MLV was performed by reverse transcription followed by PCR. The activity was confirmed in *V. natriegens* PF and *E. coli* BL21 (Figure 11). Quantification of reverse transcriptase activity by real-time PCR is planned. However, Taq DNA polymerase lacked enzymatic activity despite precipitation in downstream processes. Our efforts to obtain a functional sample of Taq DNA polymerase were unsuccessful, suggesting the need for further optimization.

## 4. Discussion

*E. coli* remains the most widely used host organism in the production of recombinant proteins. Several approaches have been developed to improve protein production, such as codon optimization [34], the screening and creation of new strains [1], and the use of tags or chaperones [35]. Nevertheless, a lack of protein production, the formation of inclusion bodies [36], or the contamination of the culture with phages [37] often occurs. Therefore, the use of an alternative expression system, such as the Gram-negative bacterium *V. natriegens*, is advisable. It has a doubling time of <10 min [2], which makes it the fastest useable growing bacterium and thus a highly attractive expression host [5]. The explanation for the high levels of biosynthesis might lie partly with its number of ribosomes since *V. natriegens* has 115,000 per cell in the exponential phase, whereas *E. coli* has 70,000–90,000 [38].

The primary objective of our study has been to evaluate the production efficiency of mutant Taq DNA polymerase and mutant M-MLV reverse transcriptase under controlled conditions in two host organisms: *E. coli* and *V. natriegens*. Another goal has been to determine the optimal organism for the production of these specific proteins. Additionally, our aim has been to assess whether different expression conditions, including media composition and temperature, influence the yield of the target proteins.

The selection of a suitable culture medium is an important factor for protein production, with several essential components such as a carbon source, nitrogen, essential salts, and minerals being required. In general, three types of media are available: chemically defined, semi-defined, and complex. The selection of the appropriate medium is important to achieve optimum cell density. Changing the conditions of cultivation is the simplest modification for altering bacterial growth. The most suitable medium for *E. coli* is LB medium and a simple change to richer media can lead to an increase in production [39]. Media change was successful in the case of the Taq DNA polymerase when its production in *E. coli* BL21(DE3) cells increased from 21% of TCPs in LB medium to 26% of TCPs in TB medium at 37 °C. M-MLV reverse transcriptase production was comparable in both media at 37 °C and accounted for approximately 16% of TCPs. *V. natriegens* is a marine bacterium, so it needs an increased salt concentration for optimal growth. Lee et al. [4] used LB medium with the addition of 3% NaCl and standardized LB3 medium as a medium suitable for *V. natriegens* because of its simplicity. Xu et al. [7] employed a modified TB medium with the addition of 1.5% NaCl (designated here as TB1.5). We utilized these two media in *V. natriegens* as complementary to the media in which we grew *E. coli*. However, the use of the richer TB1.5 medium did not increase the production rate, as the Taq DNA polymerase production rate dropped from 30% to 19% of TCPs (LB3 versus TB1.5), and similarly for MMLV reverse transcriptase, from 11% to 6% of TCPs.

Protein misfolding is a significant challenge in the bacterial production of recombinant proteins [40]. The lowering of the expression temperature is typically one of the first and easiest steps to increase the solubility of the produced protein [41,42]. The use of lower temperatures in production processes usually results in higher yields of soluble protein and improved biological activity of the protein [43]. However, in all our cases, no obvious benefit arose from a reduction of expression temperature. Our results indicate a significant effect of temperature on the yield of the expression system. In the case of the production of mutant M-MLV reverse transcriptase in *E. coli* BL21, a 2.5-fold decrease occurred in the percentage representation of the target protein at a lower temperature in both media. During the production at lower temperatures in *V. natriegens* PF, a more significant reduction was detected in yield compared with production in *E. coli*. In LB medium, the yield decreased by 4.4-fold, and in TB medium by 3-fold. Interestingly, for mutant Taq DNA polymerase production, as expected, a decrease was also found in production, but unlike in the case of mutant M-MLV reverse transcriptase production, the decrease was higher in *E. coli* than in *V. natriegens*. Regarding production in *V. natriegens* Vmax™ Express, a smaller decrease was recorded in production at lower temperatures compared with production in *E. coli*, in which protein production decreased in LB medium by 1.6-fold and in TB medium by 1.9-fold. For the production of mutant Taq DNA polymerase in *E. coli* BL21(DE3), the production decrease was 2.2-fold in LB medium and 2.6-fold in TB medium. The decrease in production temperature had no impact on protein solubility in any of the cases. Given the low yields at lower temperatures, we assessed production at lower temperatures as being less favorable. The variance in our results might arise not only from the target protein but also from the utilization of distinct strains of the same organisms. Another factor to consider is that even single-point mutations can cause significant changes in protein solubility [44].

Additionally, since lowering the temperature of expression did not help to increase the solubility of the target protein in any of the cases in our study, we focused on increasing the solubility by using various sonication buffers. Importantly, when selecting a suitable sonication buffer, not only should the host organism be considered, but also the protein being produced whose properties, e.g., protein activity, might be affected. Our results show, in both cases, the importance of the determination of a suitable buffer. Regarding the evaluation of the solubility of M-MLV, our findings distinctly demonstrate a higher production of the protein in *E. coli* in the soluble fraction. Interestingly, in the case of production in *E. coli*, we noticed relatively large differences in the effect of the sonication buffer on the solubility of M-MLV in comparison to *V. natriegens*. Based on our findings, Buffer A is the most suitable option for M-MLV production in *E. coli*, yielding 1.6 times more soluble protein compared with that in PBS buffer and 2 times more than that in Buffer B. The highest solubility was attained in the PBS buffer, but in comparison with the lowest solubility achieved in Buffer B, this value was only 1.3 times higher. Interestingly, we obtained nearly opposite results when assessing the solubility of Taq pol. We detected only a negligible amount of soluble protein detected by Western blot when produced in *E. coli*. On the other hand, we were able to obtain 23% of TCPs when using Buffer B, which is 1.3 times higher than the lowest amount of protein obtained when using Buffer A. We did not observe a significant difference in the amount of soluble protein when using Buffer B compared with PBS.

We were only able to produce negligible amounts of soluble Taq polymerase in *E. coli* intracellularly. *V. natriegens* intracellular production resulted in increased solubility when we used diverse buffers. Extracellular protein production can be an excellent strategy for producing recombinant proteins that are insoluble when produced intracellularly. Other advantages include the simplification of downstream processes, cost-effectiveness, and time-saving. Overexpression of D,D-carboxypeptidases causes the non-selective leakage of proteins into the media [15]. Interestingly, extracellular production of Taq polymerase without co-expression after 24 h was 1.13 times higher compared with extracellular production with co-expression. The extracellular protein production without co-expression was probably caused by the induction of a prophage naturally present in *V. natriegens*. *V. natriegens* contains two prophage regions (VNP1 and VNP2) that can exhibit spontaneous activation under standard cultivation conditions [30]. The higher amounts of protein produced extracellularly without co-expression might be caused by the overall better production without co-expression, as it imposes less burden on the cell. These results suggest that extracellular production might be advantageous for the production of mutant Taq DNA polymerase; however, activity verification will be necessary. On the contrary, for extracellular M-MLV production, this strategy did not prove to be successful. This may be attributable to such production involving the passive transport of proteins across the membrane without any secretion signals or to low production overall. Nevertheless, extracellular production might be suitable for proteins such as enzymes, as it allows the shortening and simplification of downstream processes, eliminating the need for harsh cell homogenization and similar steps that might affect the activity of the produced enzyme.

M-MLV reverse transcriptase activity was confirmed in both expression systems, so we were able to produce an active enzyme. The activity was determined by reverse transcription followed by cDNA amplification. In our case, RNA for GFP protein was used, a similar procedure for qualitative assay having been chosen by Nuryana et al. [45] when they used RNA of SARS-CoV-2 as a template. Subsequently, they also determined the specific activity of the enzyme; for the quantitative assay, the reverse transcriptase activity was measured using an EnzChek^TM^ RT assay kit. We will focus on the quantification of synthesized cDNA by using qPCR and other quantification methods in future work. We have not been able to obtain an active Taq DNA polymerase as yet. The inactivity of the enzyme might be caused by the protein precipitation method or by the degradation of the protein itself. Chen et al. have found that ammonium sulfate precipitation leads to the loss of Taq Polymerase I activity by 30–40% [46]. We could probably increase the activity of mutant Taq DNA polymerase by ethanol precipitation, as originally suggested by Chen et al. [46]. The huge advantage of ethanol precipitation is that it does not require dialysis, which saves time, reduces costs, and prevents the loss of enzyme activity. Another aspect to take into consideration is the storage time. According to Chen et al., the Taq Pol Ι activity is lost after 2 days of storage at −20 °C [46]. However, we analyzed the activity of mutant Taq DNA polymerase immediately after the purification step. Nevertheless, we propose that, in our case, the loss of enzymatic activity is probably caused by the combination of the single-point mutations that were introduced into the sequence of the enzyme. According to Mildvan et al. [47], when two mutations have opposing structural effects, they can negatively impact the enzyme’s ability to bind to its substrate. If the negative impact is significant enough, it can lead to the inactivity of the enzyme. We will now focus on the gradual mutation of the enzyme and on monitoring the impact of individual mutations on the enzyme activity.

## 5. Conclusions

Our study was focused on a comparison of *E. coli* and *V. natriegens* for the intracellular production of enzymes, specifically mutant Taq DNA polymerase and mutant M-MLV reverse transcriptase. We evaluated various growth conditions, such as media types, temperature effects on protein production, and the solubility of the protein in both organisms. The solubility of the target proteins varied depending on the organism and the sonication buffer used. Part of our study was aimed at the extracellular production of these proteins in *V. natriegens* since extracellular production can be an effective strategy for producing recombinant proteins that are insoluble intracellularly. Our study has highlighted the potential of *V. natriegens* as an alternative to *E. coli* for the production of nucleic acid metabolism enzymes, as its characteristics of rapid growth, accessible molecular tools, and other advantageous properties show promise for many industrial applications.

## Figures and Tables

**Table 1:** All strains used.

Strain	Genotype	Source
*E. coli* BL21 (DE3)	B F—ompT gal dcmlonhsdSB(rB–mB–) λ(DE3 [lacI lacUV5T7p07 ind1 sam7 nin5]) [malB+]K-12(λS)	New England Biolabs^®^ Ipswich, Ipswich, MA, USA
*E. coli* BL21	F—ompT gal dcm lon hsdSB(rB–mB–) [malB+]K-12(λS)	New England Biolabs^®^
*V. natriegens* Vmax™ Express	Δ*dns*, insertion of IPTG-inducible T7 RNA polymerase cassette	Synthetic Genomics, Inc., La Jolla, CA, USA
*V. natriegens* PF	Δ*vnp1*, Δ*vnp2*	[30]

**Table 2:** All plasmids used.

Plasmid	Size	Genotype	Source
pET28a-mutTaqDNApol	7.8 kbp	T7 promoter; f1 ori; Kan^R^; TaqDNApol; His6	This work
pJexpress404-mutMMLV	6 kbp	T5 promoter; pUC ori; Amp^R^; M-MLV; His6	This work
pRSFDuet-T5-PBP5/6	6 kbp	T5 promoter; RSF ori; Cm^R^; PBP5/6	[16]
pRSFDuet-T7-PBP5/6	6 kbp	T7 promoter; RSF ori; Cm^R^; PBP5/6	[16]

**Table 3:** All buffer solution compositions used.

Sonication Buffer	Composition
Buffer A	50 mM TrisHCl pH 8, 0.5 M NaCl, 15% glycerol
PBS	137 mM NaCl, 10 mM Na_2_HPO_4_, 2.7 mM KCl, 1.8 mM KH_2_PO_4_
Buffer B	50 mM TrisHCl pH 8, 1 mM DTT, 1 mM EDTA, 1 mM PMSF

## Data Availability

All data generated or analyzed during this study are included in this published article.
